# Harvesting a 3D N-Doped Carbon Network from Waste Bean Dregs by Ionothermal Carbonization as an Electrocatalyst for an Oxygen Reduction Reaction

**DOI:** 10.3390/ma10121366

**Published:** 2017-11-28

**Authors:** Yimai Chen, Hui Wang, Shan Ji, Weizhong Lv, Rongfang Wang

**Affiliations:** 1 College of Biological, Chemical Science and Chemical Engineering, Jiaxing University, Jiaxing 314001, China; 15900000279@163.com; 2 College of Chemistry and Chemical Engineering, Northwest Normal University, Lanzhou 730070, China; wanghui3931@126.com; 3 Institute of Chemical Engineering, Qingdao University of Science and Technology, Qingdao 266042, China; 4 College of Chemistry and Environmental Engineering, Shenzhen University, Shenzhen 518060, China; weizhonglv@163.com

**Keywords:** ionothermal carbonization, biomass, N-doped carbon network, non-noble metal catalysts, oxygen reduction reaction

## Abstract

Three-dimensional nitrogen-doped carbon (3D-NCN) has been synthesized via the ionothermal carbonization method using waste soybean dregs (SD) as the precursor. N_2_ adsorption/desorption isotherms show that the as-prepared 3D-NCN formed a hierarchically porous structure with a specific BET surface area of 1093.4 m^2^ g^−1^ and a total pore volume of 1.77 cm^3^ g^−1^. The TEM images clearly show that graphene-like carbon sheets were formed on the edge of the networks. The characterization of the samples collected at different temperature indicated that salt melt plays the key role in the formation of the network structure and rich pores. When 3D-NCN is as electrocatalyst for ORR, it shows an onset potential of 0.945 V with a more positive half-wave potential (0.846 V), which is comparable to that of commercial Pt/C. In addition, the long-term cycle results show that the onset potential and half-wave potential only negatively shifted by 6 mV and 8 mV after 10,000 cycles respectively, which are smaller than those values of commercial Pt/C. Due to its high ORR activity, durability, and low-cost, producing 3D-NCN from SD in molten salt medium provides a promising approach to replace the Pt-based catalysts for use in fuel cells.

## 1. Introduction

Due to the global warming and environmental pollution mainly resulting from using fossil fuels as energy sources, it is currently imperative for human beings to shift to clean energy [1,2]. Among these new technologies for clean energy, fuel cells and metal-air batteries play an important role in a variety of clean energy applications. However, their practical applications are impeded by the sluggish kinetics of the oxygen reduction reaction (ORR) at the cathode. So far, Pt-based electrocatalysts are still the most active and durable ORR catalysts, but the scarcity of Pt supply, poor durability, undesirable cross-over effect, and high cost of Pt-based catalysts are major bottlenecks in the applications of these clean energy sources at a large scale [3]. With the development of fuel cell and metal-air batteries, there is a vital need for efficient and affordable nonprecious metal or metal-free ORR catalysts [4].

Currently, nitrogen-doped carbon materials are widely recognized as a promising alternative to the Pt-based ORR catalyst to achieve a low-cost and high durability without sacrificing ORR activity [5,6]. Abundant π electrons in carbon are inert to ORR, but these π electrons can be activated by doping nitrogen due to its strong electronegativity, which can favor the adsorption of O_2_ molecules on the strong positive charge of C atoms created by adjacent N atoms, and further lead to the improved stability, conductivity, and durability of carbon in the electrocatalytic processes [7,8]. Consequently, a lot of work has been conducted to modify the carbon materials with N atoms to improve the electrocatalytic performance of ORR. Usually, the N-doped carbon can be prepared via two different routes: (1) pyrolyzing the carbon materials with nitrogen containing compounds, such as NH_3_ [9]; (2) heat-treating the precursors containing nitrogen, for instance, melamine [10] and pyridine [11]. Recently, it has also been found that the direct pyrolysis of biomass provides an effective and low-cost strategy to produce N-doped carbon since there are many nitrogen-containing compounds in the biomass, such as proteins and amino acids [12,13,14]. Many N-doped carbons have been made from biomass and show a promising electrocatalytic performance for ORR. For instance, Wang et al. [15] developed a facile method to prepare an N-doped carbon ORR catalyst from chicken bones, and the obtained N-doped carbon has a high specific surface area. Compared to commercial Pt/C (10 wt %), the N-doped carbon derived from chicken bone exhibits comparable electrocatalytic activity, long stability, and excellent methanol tolerance. N-doped carbon sheets with a high nitrogen content and high specific surface area have been synthesized by using okara as both carbon and nitrogen sources [16]. Xu et al. [17] reported a method to prepare N-doped carbon using chrysanthemum as a precursor, and the obtained N-doped carbon exhibits relatively high ORR activity with better methanol tolerance and stability than that of commercial Pt/C. However, the electrocatalytic activity of these N-doped carbons still needs to be improved to meet the requirements of practical applications in fuel cells and metal-air batteries. 

Developing mesoporous N-doped carbons with a high specific surface area and various pore diameters has been considered an effective way to further improve the ORR activity of N-doped carbon due to their unique electronic and structural properties [18]. To a large extent, the properties of mesoporous N-doped carbon are strongly dependent on the porosity and specific surface area [19]. Recently, great efforts have been contributing to the development of mesoporous N-doped carbons. Usually, this includes using hard or soft templates is the primary approach to prepare these N-doped carbons with mesopores and a high surface area. SBA-15 [20], silica nanoparticles [19,21], and MCM-48 [22] are commonly used as templates. It was also reported that an ultra-high surface area N-doped carbon could be synthesized by carbonizing biomass, such as sheep bones [6], with KOH at a high temperature. The specific surface area of the obtained carbon is up to 1961 m^2^ g^−1^, which provides rich active sites for ORR. Unfortunately, in most cases, some extremely corrosive chemicals are required to remove the templates after the N-doped carbon is formed or to use KOH to form a high specific surface area, which make these mesoporous N-doped carbons difficult to scale up for commercial applications. If mesoporous N-doped carbons with a tunable porosity and high surface area could be produced from biomass under mild conditions, it would be of great significance for commercial applications of mesoporous N-doped carbons.

Considering the above issues, we develop an ionothermal carbonization method to synthesize biomass-derived three-dimensional N-doped carbon networks (3D-NCN) with a tunable porous structure to avoid using extremely corrosive chemicals. The ionothermal carbonization provides a facile, versatile, and low-cost strategy to generate nano-sized materials at a relatively low temperature [23], and also results in a shorter reaction time with a little residual impurity compared to conventional pyrolysis processes. The electrocatalytic results exhibited that the SD-derived 3D-NCN shows high ORR activity, comparable to commercial Pt/C in terms of activity and durability.

## 2. Experimental

### 2.1. Synthesis of Mesoporous N-Doped Carbon

3D-NCN was prepared as follows: SD were first dried at 80 °C in an oven for four days, and then the dried SD was sifted through a 80 mesh sieve to remove the big particles. A total of 1 g of dried SD was mixed with 15 g of NaCl/ZnCl_2_ mixture and ball milled in a zircon jar at 600 rpm for 6 h. After that, the mixture was loaded into a ceramic crucible and then placed in an inert gas tube furnace. The temperature was elevated to 900 °C with a heating rate of 2.5 °C min^−1^ under nitrogen atmosphere, and kept at this temperature for 60 min. Subsequently, the temperature was cooled down to 50 °C with a cooling rate of 5 °C min^−1^. The obtained product was immersed into 400 mL of H_2_O and magnetically stirred for 6 h, then filtered out and dried at 80 °C in a vacuum oven for 12 h. Afterwards, the carbon material was immersed in 2 mol L^−1^ HNO_3_ solution for 24 h to remove inorganic insoluble components, if any were present. After acid washing, the sample was washed with water until the pH of the filtered water was neutral, and was then dried at 80 °C in a vacuum oven for 12 h. Afterwards, 1 g of 3D-NCN with 1 g of FeCl_3_ was dissolved in 5 mL of water, dried at 60 °C for 12 h, and then heated at 800 °C for 2 h. The obtained product was treated with HNO_3_ to remove unstable Fe species. The sample was rinsed with water until the pH of the filtrate was neutral, and dried at 60 °C for 12 h. 

### 2.2. Physical Characterizations

X-ray diffraction (XRD) patterns were performed on Shimadzu XD–3A with CuKα radiation (Shimadzu, Kyoto, Japan). The scan was conducted between 5° and 80° with a rate of 4° min^−1^ for 2θ values. The morphology of the carbon samples was studied by scanning electron microscopy (SEM, Carl Zeiss, Oberkochen, Germany) and transmission electron microscopy (TEM, JEOL Co., Kyoto, Japan). The SEM filming was on a Carl Zeiss Ultra Plus instrument. TEM and high angle annular dark field scanning transmission electron microscopy (STEM) images of the catalysts were produced using a JEOL (JEM-2000 FX)(JEOL Co., Kyoto, Japan) microscope operating at 200 kV. The specific surface area was determined by the nitrogen adsorption region, and the total pore volume was measured at 0.99. Furthermore, the pore size distribution was obtained with the density functional theory (DFT) method. X-ray Photoelectron Spectroscopy (XPS) spectra were generated with a PHI-5702 multifunctional X-ray photoelectron spectrometer (Physical Electronics, San Francisco, CA, USA). Binding energies were calibrated by referencing to the C 1 s peak at 285.0 eV. The amount of metal elements was analyzed by Agilent 7700(Agilent Technologies Inc., Sydney, Australia). Raman spectroscopy was recorded on a Ft-Raman spectroscope (RFS 100, BRU-KER, Karlsruhe, Germany) with a 1064 nm excitation laser beam wavelength. The intensity of the D and G peaks was gauged by the height from the top point of each band.

### 2.3. Electrochemical Characterizations

The ORR activities were performed using CHI 650D (CH Instruments) with a glass carbon (GC) electrode of a diameter of 5 mm as the working electrode, a Pt wire as the counter electrode, and Ag/AgCl (saturated KCl) as the reference one. The GC electrode was polished before every test. Each carbon suspension was prepared by dispersing 2 mg activated carbon in 0.4 mL Nafion/ethanol (0.25% Nafion) solution. After sonication for 20 min, 10 μL of the ink was applied to the GC electrode, and then dried under room temperature. The geometric area of the working electrode was about 0.196 cm^2^, and the mass loading of each carbon material, as well as commercial 20 wt % Pt/C, was about 0.255 mg cm^−2^. High-purity N_2_ or O_2_ should be saturated when the ORR test is run. The cyclic votammetry (CV) was scanned between −1 V and 0.2 V (vs*.* Ag/AgCl) with 50 mV s^−1^. During linear sweep voltammetry (LSV), the scan range was from 0.2 V to −0.8 V (vs*.* Ag/AgCl) with 5 mV s^−1^. The LSV current in oxygen subtracted from that of nitrogen, resulted in the LSV for ORR. For the calculation of the apparent transferred electron number, the LSV was measured at different rotation speeds, from 400 rpm to 2400 rpm. For methanol tolerance and the SCN^−^ ions coordination test in O_2_-saturated 0.1 M KOH, the potential was kept at 0.8 V (vs. RHE) with a constant rotation speed of 1600 rpm. The volume of injected CH_3_OH was ten percent of the electrolyte. KSCN was added at the 80th second during the chronoamperometric test, resulting in a 0.01 M SCN^−^ ion. For long-term stability, the continuous CV was scanned for 10,000 cycles. 

## 3. Results and Discussion

The NaCl/ZnCl_2_ mixture was chosen as the eutectic salt for this study. It was reported that the pores could be generated when the carbon source was heated with ZnCl_2_ due to the carbothermal reduction of Zn^2+^, and Zn^2+^ could also catalyze the dehydration of carbohydrate, resulting in a high yield [24]. In addition, when ZnCl_2_ was mixed with other salts, a larger amount of salt nanodroplet would form and disperse inside the carbon, and resulted in porous structures. According to the NaCl/ZnCl_2_ phase diagram provided in Figure S1 (in Electronic Supplementary Information, ESI) [25], there are two phases co-existing in the mixture (the ratio of NaCl to ZnCl_2_ is 80:20) in a broad temperature range (about 410 to 670 °C), namely eutectic molten salt and solid NaCl. When the amount of NaCl is 80 mol %, the temperature range for the co-existence of the two phases of eutectic salts and solid NaCl is broader than the NaCl/ZnCl_2_ mixture with a low NaCl content. The solid fraction of NaCl can cause mesopores and even macropores due to its separation of the precursor. Therefore, the NaCl/ZnCl_2_ mixture with 80 mol % of NaCl as molten salt medium was chosen in this study. 

**Figure 1:**
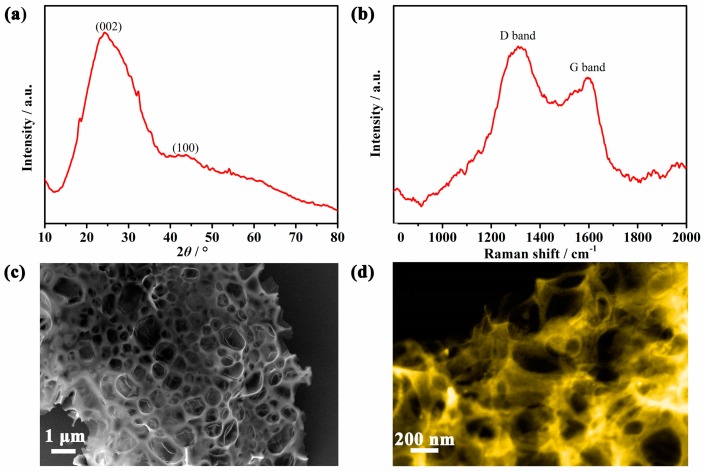
(**a**) XRD pattern; (**b**) Raman spectroscopy; (**c**) TEM image; and (**d**) STEM image of 3D-NCN.

Figure 1a is the XRD pattern of the as-prepared 3D-NCN. Two diffraction peaks at 2θ values of ca. 24° and 43° corresponding to the (002) and (100) peak, respectively, appear in the curve, which is similar to the XRD pattern of graphite. However, the two peaks are broad, indicating that the graphitization of this sample is low. This is due to relatively low pyrolysis temperature of 900 °C, which isn’t high enough to form a good graphite structure, so the carbon still has a partially disordered structure. Based on the broad (002) peak, the carbon structure could be two to threelayer-stacked sheets [26]. Raman spectroscopy was also performed to characterize the disordered structure and degree of structural defect for the as-prepared sample. As shown in Figure 1b, the peak located at around 1300 cm^−1^ shows the characteristics of the disordered carbon (D band), a symbol of internal defects. The peak at around 1600 cm^−1^ corresponds to graphite layers (G band), attributed to ideal graphitic lattice vibrations of sp^2^ carbon atoms [27,28]. The extent of the defects can be quantified by the ratio of the *I*_D_*/I*_G_ in graphite materials; the higher the *I*_D_*/I*_G_ ratio is, the lower the crystallinity is [6]. The *I*_D_/*I*_G_ value of 3D-NCN is 1.13, which is slightly higher than those of the reported homemade carbon [2,15,29,30], implying that there are more edges and defects in the obtained 3D-NCN, which could result in more active sites for electrocatalytic reactions. As shown in Figure 1c, a unique porous network, namely a very open morphology with quite thin walls, was formed. The STEM image (Figure 1d) further exhibits an interconnected network of submicrometersized macropores in the as-prepared 3D carbon material.

**Figure 2:**
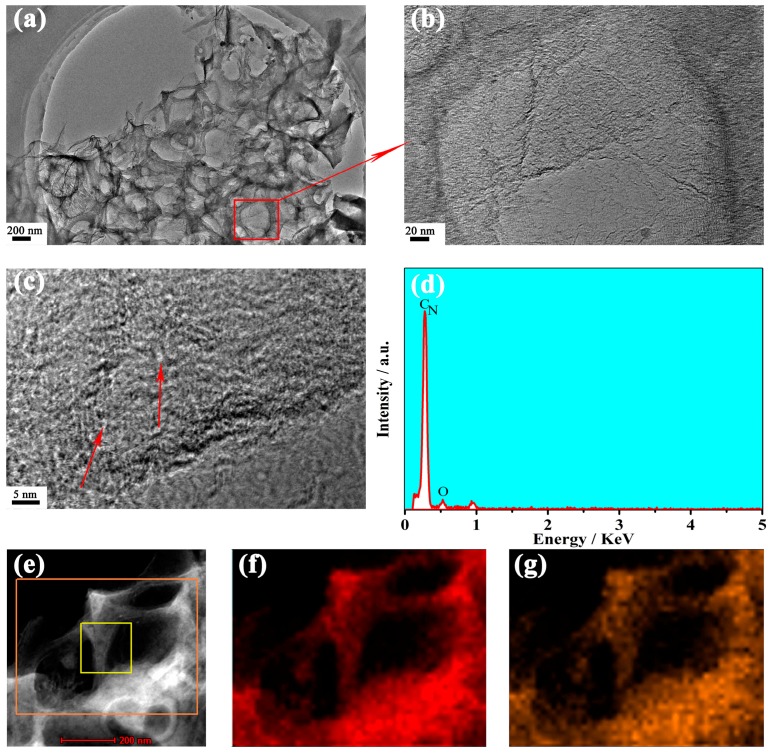
(**a**–**c**) TEM images with different magnification; (**d**) EDX; (**e**) STEM image, and elemental mapping of (**f**) C and (**g**) N of 3D-NCN.

The TEM picture of the sample displayed in Figure 2a confirmed that the open porous structure was formed in the as-prepared carbon. The distribution of these large pores is uniform, whose size is in the range of tens to hundreds of nanometers. The enlarged picture of the selected area in Figure 2a,b shows that the carbon walls on the edges of the large pores are extremely thin graphite layers. Meanwhile, the large number of micropores indicated in Figure 2c by the red arrows appear in the carbon walls, which could lead to a highly specific surface area and rich active sites forming on its surface. The EDX result in Figure 2d shows that 3D-NCN was composed of nitrogen, oxygen, and carbon elements. The element distribution was verified in the selected region in Figure 2e. As shown in Figure 2f,g, the C and N elements are uniformly distributed on the surface, indicating that the 3D-NCN has a homogeneous structure. 

**Figure 3:**
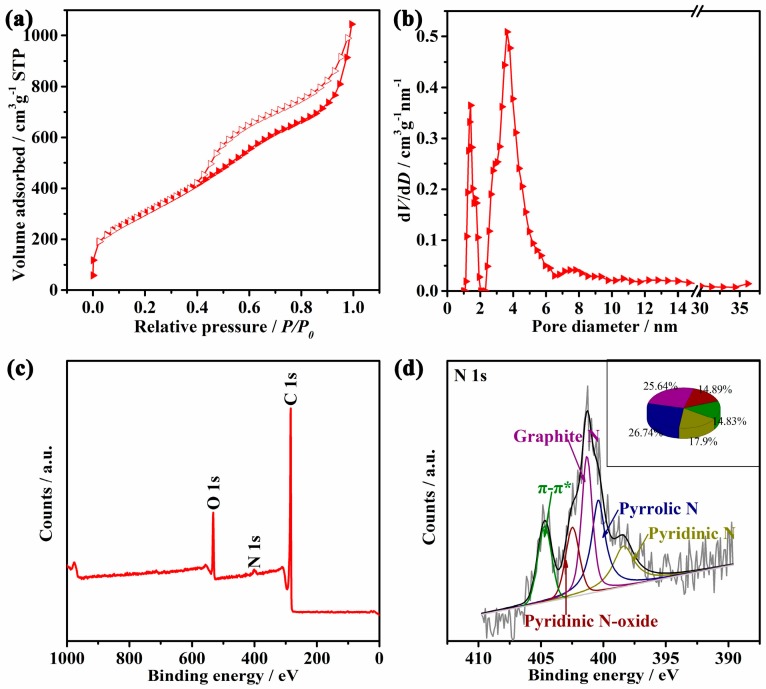
(**a**) N_2_ isotherm; (**b**) pore size distribution; (**c**) XPS survey spectrum and (**d**) N 1s spectrum of 3D-NCN.

An N_2_ adsorption-desorption isotherm was carried out to gain a deep insight into the porous structure of the as-prepared 3D-NCN. The N_2_ isotherm of 3D-NCN shown in Figure 3a is of type IV with a high N_2_ uptake in a low relative pressure region and hysteresis in a high relative pressure region, suggesting that the sample contained both microporous and mesoporous structures. In addition, there is rapid N_2_ adsorption at a high relative region (P/P_0_ > 0.9), which is attributed to the macropores in the 3D-NCN [31]. The pore size distribution of the 3D-NCN in Figure 3b clearly shows two peaks at ca. 1.4 and 3.7 nm, respectively, and most of its pore size distribution is in a relatively narrow range of 1.0 to 6.0 nm. The results indicate that the 3D-NCN possesses a hierarchically porous structure from micro to macropores. The macropores can act as a bulk buffer for storing electrolytes and further shorten the mass diffusion paths to the interior surface of pores in the smaller pore size region [32]. The mesoporous structure more easily provides an accessible surface area for gaseous activation molecules than conventional carbon materials, so it is expected that the 3D-NCN with a high number of mesopores would exhibit a better electrocatalytic performance. The specific surface area of the sample calculated via the Brunauer-Emmett-Teller model is 1093.4 m^2^ g^−1^, and the total pore volume is 1.77 cm^3^ g^−1^. 

The XPS measurement was carried out to investigate the sample’s surface composition (within a range of 0.1–10 nm) and elemental chemical states [33]. Figure 3c is the XPS survey spectrum of 3D-NCN, and shows clear signals of C 1s, O 1s, and N 1s, agreeing with the EDX result. Fe, Na, and Zn signals are not observed from the XPS survey spectrum, indicating that these atoms on the surface were removed. As illustrated in Figure S2a (ESI), the carbon species on the surface of 3D-NCN can be fitted into three peaks, namely sp^2^ C=C, sp^3^ C–C, and C=O at 284.8 eV, 286.1 eV, and 288.9 eV, respectively. It can be seen that the majority of the carbon on the surface of the sample is sp^2^ carbon. The high amount of sp^2^ carbon could improve the electron conductivity, which could facilitate electron transfer during the electrocatalytic processes [34]. As shown in Figure S2b (ESI), there are two types of oxygen on the surface, namely C–O (–O–) located at ca. 532 eV and O=C–OR at ca 533 eV, indicating the presence of oxygen-containing functional groups on the 3D-NCN surface. The high-resolution spectrum of N 1s of 3D-NCN (Figure 3d) shows five individual nitrogen states, i.e., pyridinic-N at 397.8 eV, pyrrolic-N at 399.4 eV, graphitic-N at 401.0 eV, pyridinic N-oxide at 401.9 eV, and the π–π* satellite at 404.8 eV [35,36]. A detailed percentage of these N species in the 3D-NCN is illustrated in the Inset. Among these N species, pyridinic-N provides a pair of electrons to bond with the *p*-conjugated rings, and the pyrrolic-N is good at donating electrons, which can improve the electrochemical performance [37,38]. Graphitic-N, especially that on the edge, has been proved to bring about a lower over-potential for ORR, resulting in a better performance [39]. The nitrogen atomic content is around 1.2 at %, and the graphitic-N is 25.64%. Recently, it has been reported that trace (ppm) levels of metallic impurities have profound influences on the observed ORR potentials [40]. Following this, the metal concentration was measured by inductively coupled plasma mass spectrometry (ICP-MS) analysis. The results show that the Fe atoms concentration is 0.05 wt %, Na 0.009 wt %, and Zn 0.01 wt %. 

**Scheme 1:**
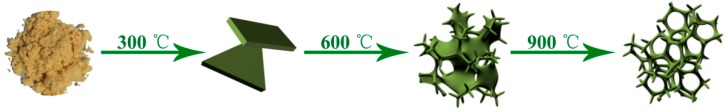
Effect of temperature on the structure of obtained carbon materials.

To gain a clear picture of the formation process of 3D networks, carbon materials were also prepared in the presence of only one salt, i.e., NaCl or ZnCl_2_. As shown in Figure S3 (ESI), irregular carbon blocks are formed instead of a network-like-structure. The pores are derived from the micro- and meso-scale (Figure S4, ESI), which is similar to that of 3D-NCN. The calculated BET surface areas are 469.1 and 768.2 m^2^ g^−1^ for NaCl or ZnCl_2_, respectively, which are obviously smaller than that of 3D-NCN. Therefore, it can be concluded that the eutectic plays a key role in the formation of the network-like-structure. At the same time, a morphological evolution study was conducted under different temperatures. The temperatures of 300 and 500 °C were selected based on the NaCl/ZnCl_2_ phase diagram. At 300 °C, there are two solid phases, NaCl and Na_2_ZnCl_4_. Blocky product was obtained at this temperature (Figure S5a,b, ESI). When the temperature was 500 °C, two phases, namely the eutectic salt and NaCl solid, coexisted. As shown in Figure S5c,d (ESI), the product appears as a fluffy shape with a few holes, indicating part of pores were formed. It could be deduced that the eutectic salt acted as the fluid template. However, the amount of porous structure formed at this temperature is low. The reason for this could be that the amount of eutectic salt formed was not enough. The specific surface areas of the carbon materials obtained at 300 °C and 500 °C are 229.7 m^2^ g^−1^ and 272.6 m^2^ g^−1^, respectively (Figure S6, ESI). The results indicate that most of the pores did not form in the presence of solid salt, suggesting again that the eutectic salt plays a vital role in the formation of a 3D network-like structure. Based on the SEM and N_2_ isotherm results, a schematic illustration of the formation of the 3D network with an increasing temperature is presented in Scheme 1. Firstly, blocky intermediates are formed below ca. 300 °C. As the temperature increases to 500 °C, the fluffy structure with a porous structure is formed. Finally, when enough eutectic salt forms, the high temperature results in the network-like nanostructure with sufficient pores. Due to the high surface area, it is expected that the 3D-NCN would exhibit a better electrocatalytic performance. 

**Figure 4:**
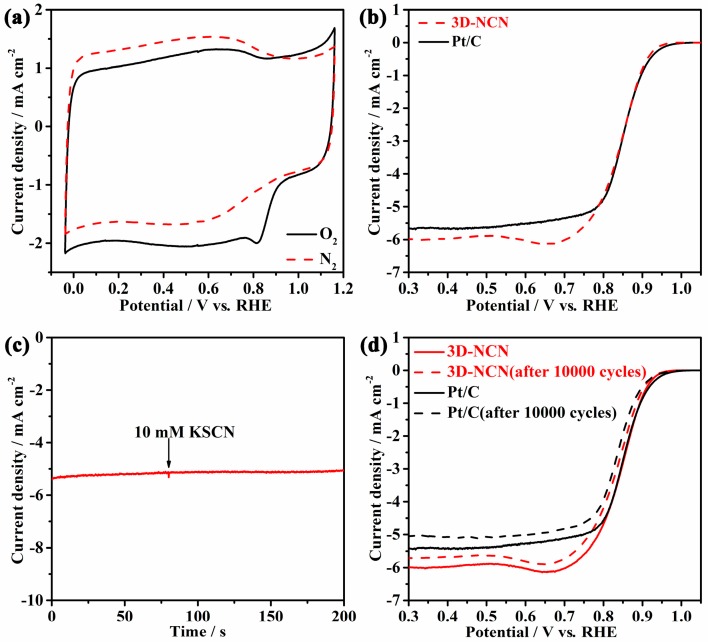
(**a**) Cyclic voltammograms of 3D-NCN in nitrogen and oxygen-saturated 0.1 M KOH electrolyte with a scan rate of 50 mV s^−1^; (**b**) RDE polarization curves of 3D-NCN and Pt/C in oxygen-saturated 0.1 M KOH at 1600 rpm, with a sweep scan rate of 5 mV s^−1^; (**c**) Chronoamperometry test of 3D-NCN in oxygen-saturated 0.1 M KOH with SCN^−^ in the solution; (**d**) Linear sweep voltammograms of 3D-NCN and Pt/C tested at 1600 rpm in O_2_-saturated 0.1 M KOH before and after 10,000 potential cycles with a sweep scan rate of 5 mV s^−1^.

Figure 4a shows the cyclic voltammograms of the as-prepared 3D-NCN sample in nitrogen- and oxygen-saturated 0.1 M KOH solutions with a scan rate of 50 mV s^−1^. The CV in the nitrogen-saturated solution show typical characteristics of N-doped carbon tested in nitrogen-saturated KOH solution, namely reversible and featureless curves with a wide voltammetirc response [6]. The wide voltammetric response results from the capacitive charging current due to the high surface areas [41]. In oxygen-saturated KOH solution, the CV curves of the as-prepared 3D-NCN samples exhibit an ORR peak at an RHE potential of 0.82 V, suggesting that it is catalytically active toward ORR. 

The ORR activity of the as-prepared N-doped carbon was evaluated by linear sweep voltammetry (LSV) in an oxygen-saturated 0.1 M KOH electrolyte (Figure 4b). For the ORR evaluation of the samples, LSVs in N_2_-saturated KOH solution were recorded to generate capacitive currents, and all LSVs in O_2_-saturated KOH solution were then subtracted from the obtained capacitive currents. The potential corresponding to 0.1 mA cm^−2^ was set as the onset potential (*E*_onset_), the current value at 0.3 V (vs. RHE) was employed as the limiting diffusion current [26], and the half wave potential (*E*_1/2_) used to represent the electrocatalytic performance was the potential at half current of the limiting diffusion current. For comparison, commercial Pt/C (20 wt %) was also tested. In the case of 3D-NCN, the onset potential is 0.945 V with an *E*_1/2_ of 0.846 V, which is comparable to those of commercial Pt/C, (its *E*_onset_ and *E*_1/2_ are 0.962 V and 0.850 V respectively), indicating that 3D-NCN has high catalytic activity for ORR due to its unique 3D porous structure.

To further evaluate the ORR kinetics, polarization curves at different rotation speeds from 400 rpm to 2400 rpm were also performed in a KOH electrolyte. Polarization curves for ORR in O_2_ saturated 0.1 M KOH solution on 3D-NCN and Pt/C electrodes at various rotation speeds are illustrated in Figure S7a (ESI). Based on the Koutecky-Levich (K-L) theory, the apparent electron transfer number (*n*) has been calculated [42]. Figure S7b (ESI) displays the K-L fitting plots, along with the ideal case of *n* = 2 and *n* = 4. At 0.462 V (vs. RHE), the electron transfer number per oxygen is 3.78 for 3D-NCN, very close to the Pt/C (around 3.8). This finding indicates that oxygen was directly reduced to OH^−^ via an efficient four-electron transfer reaction on 3D-NCN, which is similar to the effective oxygen reduction process occurring on the highly active commercial Pt/C electrode. A higher *J*_K_ means smaller kinetic limitations during ORR, usually resulting from heteroatom-doping, more open active sites, and a high surface area. To validate the origin of the superior ORR activity, SCN^−^ ions have been added to the alkaline electrolyte. SCN^−^ ions can strongly coordinate and poison Fe-based catalytic sites even in a trace amount. KSCN was added as 0.01 M SCN^−^ in the 0.1 M KOH electrolyte at the 80th second of the chronoamperometry testing. As depicted in Figure 4c, there is no obvious current loss after introducing KSCN into the KOH electrolyte. These results indicate that the ORR active sites on the surface of 3D-NCN resulted from the active N atoms on the surface of 3D-NCN.

The long-term stability is another very important parameter for commercializing these low-cost ORR catalysts [43]. The ORR durability of 3D-NCN and Pt/C was evaluated by continuous cyclic voltammetry scanning from −1 V to 0.2 V (vs. Ag/AgCl) in 0.1 M KOH for 10,000 cycles. After that, the polarization curve of the 10,000th cycle in the oxygen-saturated electrolyte was recorded and compared to the first one. Figure 4d shows that the *E*_onset_ of 3D-NCN is 6 mV more negative than that of the first cycle, and the *E*_1/2_ has negatively shifted by 8 mV after 10,000 cycles. While for commercial Pt/C, the shifts of *E*_onset_ and *E*_1/2_ are 16 mV and 15 mV, respectively. It clearly shows that the 3D-NCN is more durable than commercial Pt/C in the ORR operation conditions. It was reported that the hierarchically micro/mesoporous structure of N-doped carbon can improve mass diffusion [44]. 3D-NCN contains both micro and mesoporous structures, so there will be more active sites sustainably available to the oxygen during the ORR, further resulting in excellent durability for ORR.

## 4. Conclusions

Porous N-doped carbon networks with graphene-like edges have been successfully synthesized by ionothermal carbonization using SD as carbon and nitrogen sources. It was found that the mixed salts of NaCl/ZnCl_2_ can act as the solvent and pore forming agent during the calcination of SD. The resultant N-doped carbon possesses a 3D hierarchical structure with major pore size distribution centered at the relatively narrow mesoporous range. The KSCN testing shows that the improved ORR activity of the as-prepared 3D-NCN is from active N atoms on the surface. The ORR activity of 3D-NCN is comparable to commercial Pt/C, and its limiting current density is higher than Pt/C. The high electrocatalytic performance of 3D-NCN is attributed to its mesoporous structure, large surface area, and graphene-like edges. The ORR on the surface of 3D-NCN is also a highly efficient 4e^−^ pathway. In addition, the as-prepared 3D-NCN is highly stable during the continue cycling test, making it an encouraging ORR catalyst for practical applications.

## Supplementary Materials

The following are available online at www.mdpi.com/1996-1944/10/12/1366/s1, Figure S1: Phase diagram of NaCl/ZnCl_2_, Figure S2: The high resolution (a) C 1s and (b) O 1s XPS spectrum of 3D-NDC, Figure S3: SEM images of carbon materials prepared using NaCl(a) and ZnCl_2_(b,c) as medium respectively, Figure S4: N_2_ isotherm and pore size distribution of carbon materials prepared using NaCl (a,b) and ZnCl_2_(c,d) as medium respectively, Figure S5: SEM images of carbon materials obtained at (a,b) 300 °C and (c,d) 500 °C respectively, Figure S6: N_2_ isotherms and pore size distributions of carbon materials of carbon materials obtained at (a,b) 300 and (c,d) 500 °C respectively, Figure S7: (a) Polarization curves of 3D-NCN in oxygen-saturated 0.1 M KOH solution at various rotation rate, scan rate is 5 mV s^−1^. (b) Koutecky-Levich plots for 3D-NCN compared with ideal 2-electron and 4-electron processes at 0.462 V in 0.1 M KOH.
