# Supplementary material for: Harvesting a 3D N-Doped Carbon Network from Waste Bean Dregs by Ionothermal Carbonization as an Electrocatalyst for an Oxygen Reduction Reaction
Source: Materials (Basel). 2017 Nov 28;10(12):1366. doi: 10.3390/ma10121366 (PMC5744301; doi:10.3390/ma10121366)
Supplement: Supplementary file 1 [file materials-10-01366-s001.pdf]

materials

Electronic Supplementary Information (ESI)

Harvesting 3D N-doped carbon network from waste bean dregs by ionothermal carbonization as electrocatalyst for oxygen reduction reaction

Yimai Chen [1,2], Hui Wang [3], Shan Ji [1,4,*], Weizhong Lv [4] and Rongfang Wang [3,*]

1 College of Biological, Chemical Science and Chemical Engineering, Jiaxing University, Jiaxing 314001, China; 15900000279@163.com
2 College of Chemistry and Chemical Engineering, Northwest Normal University, Lanzhou 730070, China
3 Institute of Chemical Engineering, Qingdao University of Science and Technology, Qingdao 266042, China; wanghui3931@126.com
4 College of Chemistry and Environmental Engineering, Shenzhen University, Shenzhen 518060, China; weizhonglv@163.com

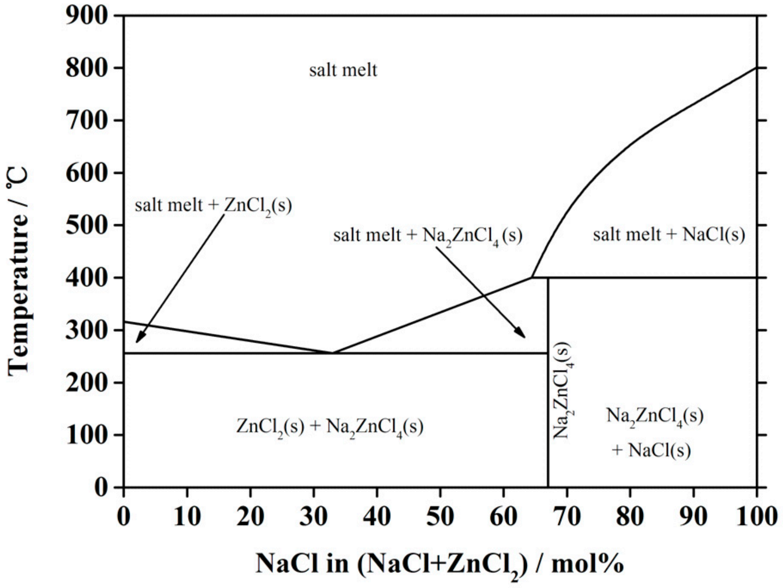

Figure S1. Phase diagram of NaCl/$ZnCl_2$.

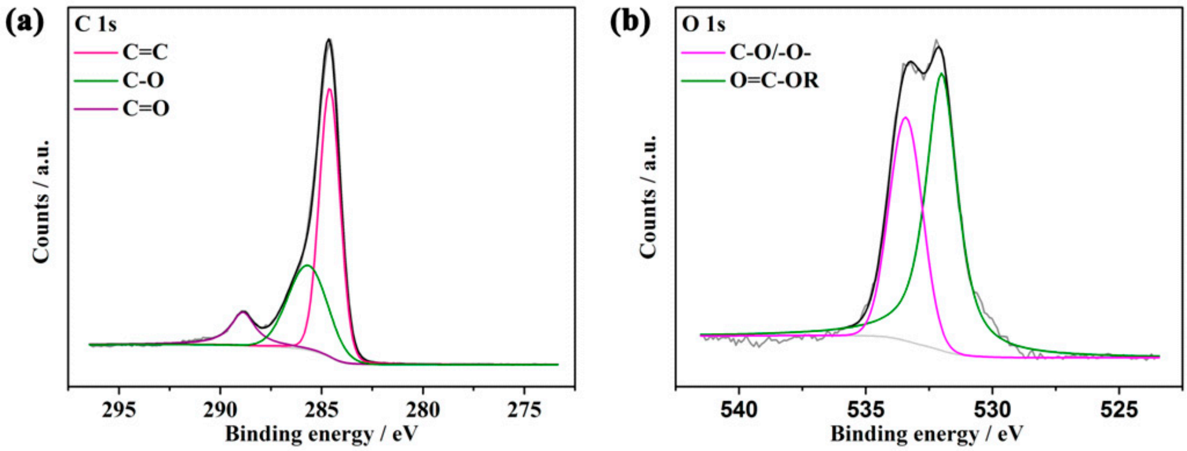

Figure S2. The high resolution (a) C 1s and (b) O 1s XPS spectrum of 3D–NDC.

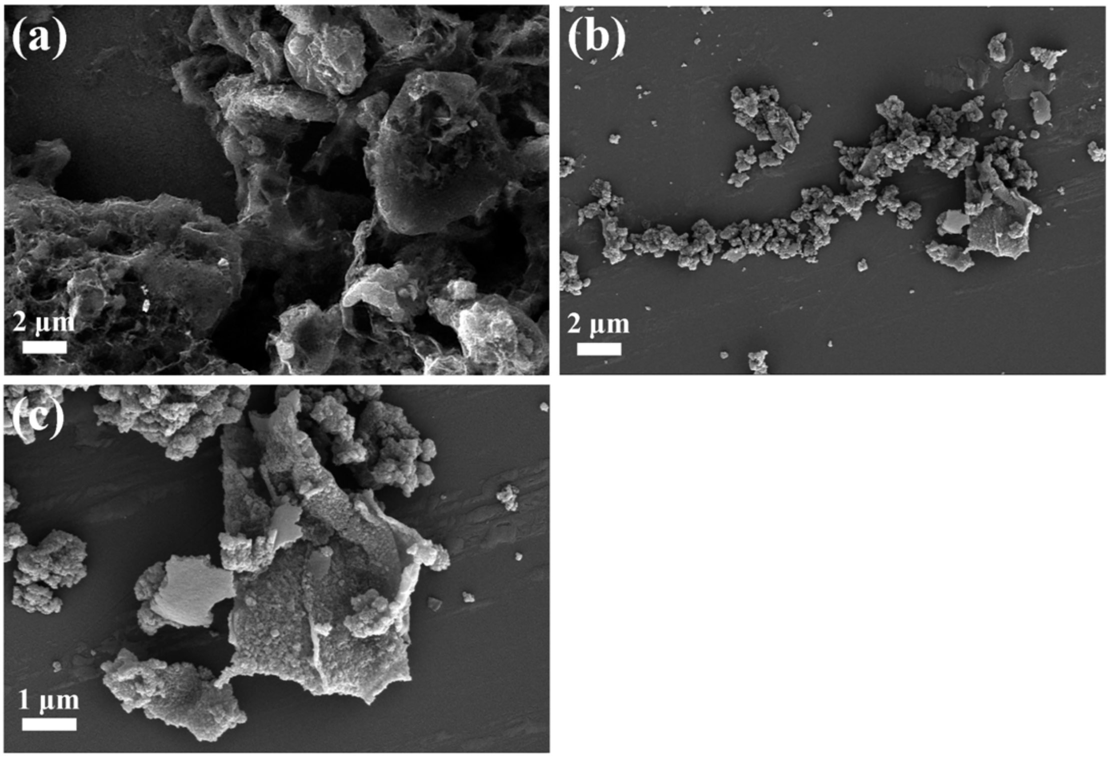

Figure S3. SEM images of carbon materials prepared using NaCl(a) and $ZnCl_2$(b,c) as medium respectively.

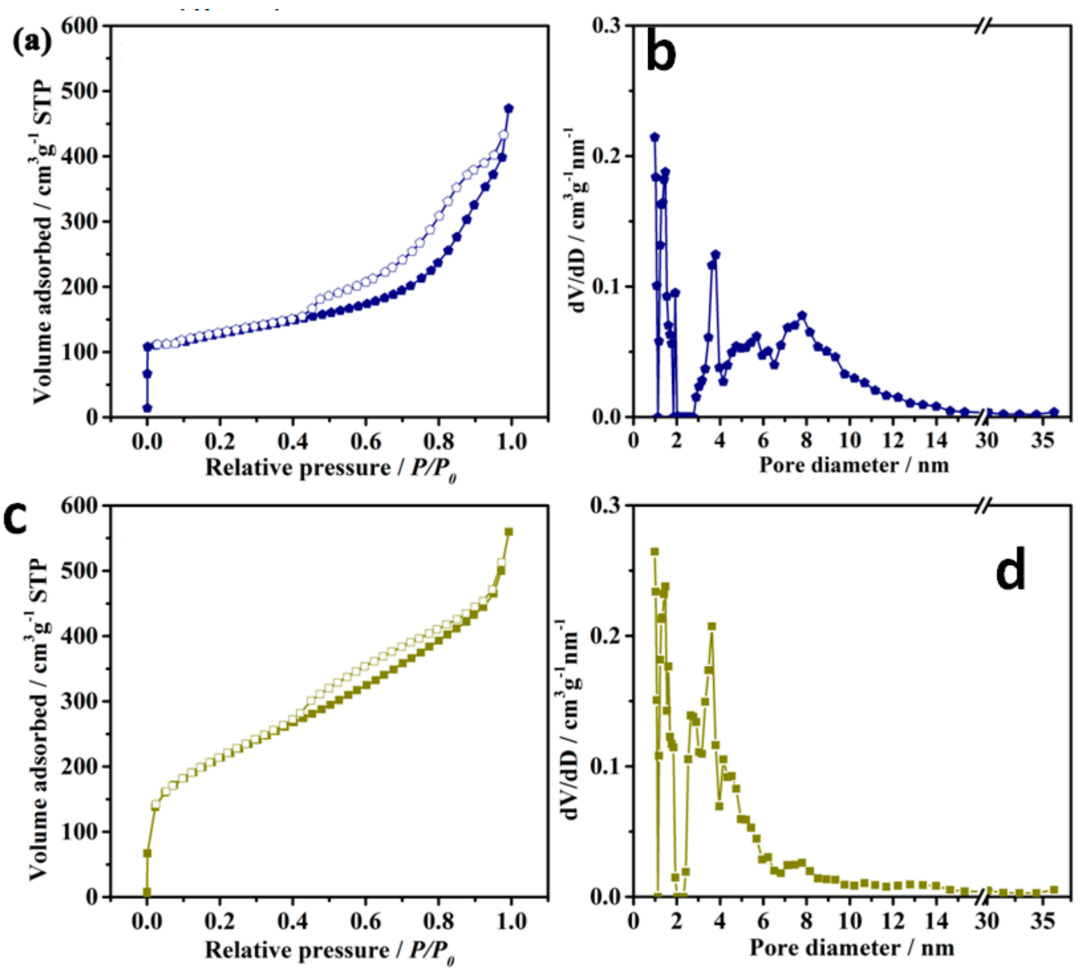

Figure S4. N_2 isotherm and pore size distribution of carbon materials prepared using NaCl (a,b) and $ZnCl_2$(c,d) as medium respectively.

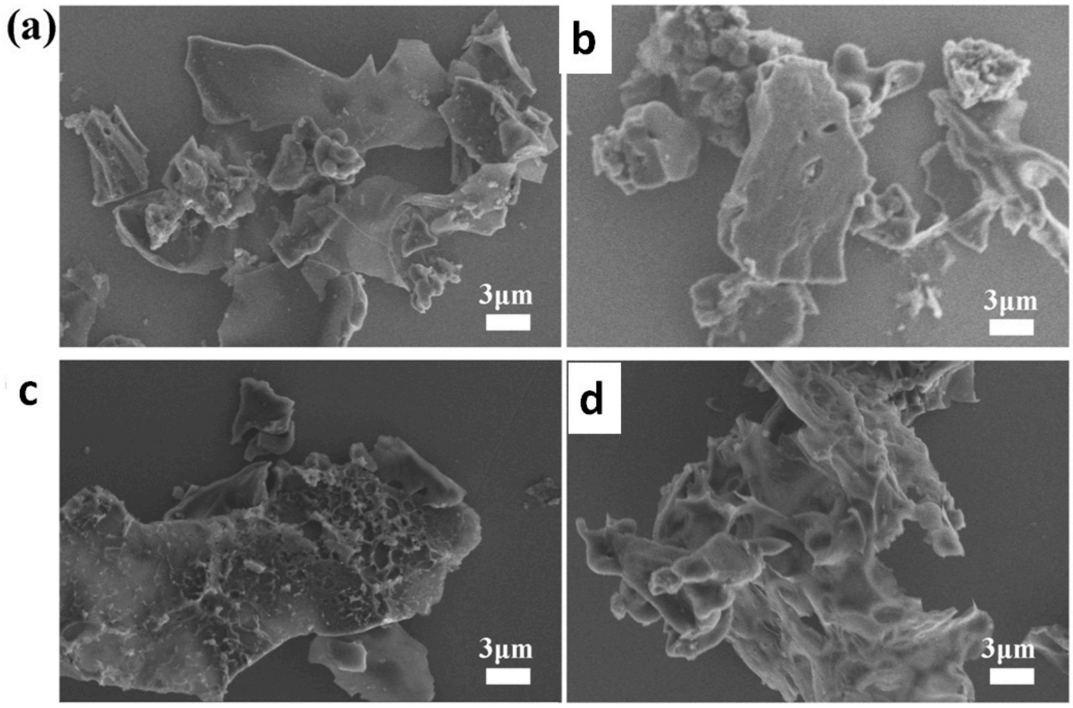

Figure S5. SEM images of carbon materials obtained at (a,b) 300 and (c,d) 500 °C respectively.

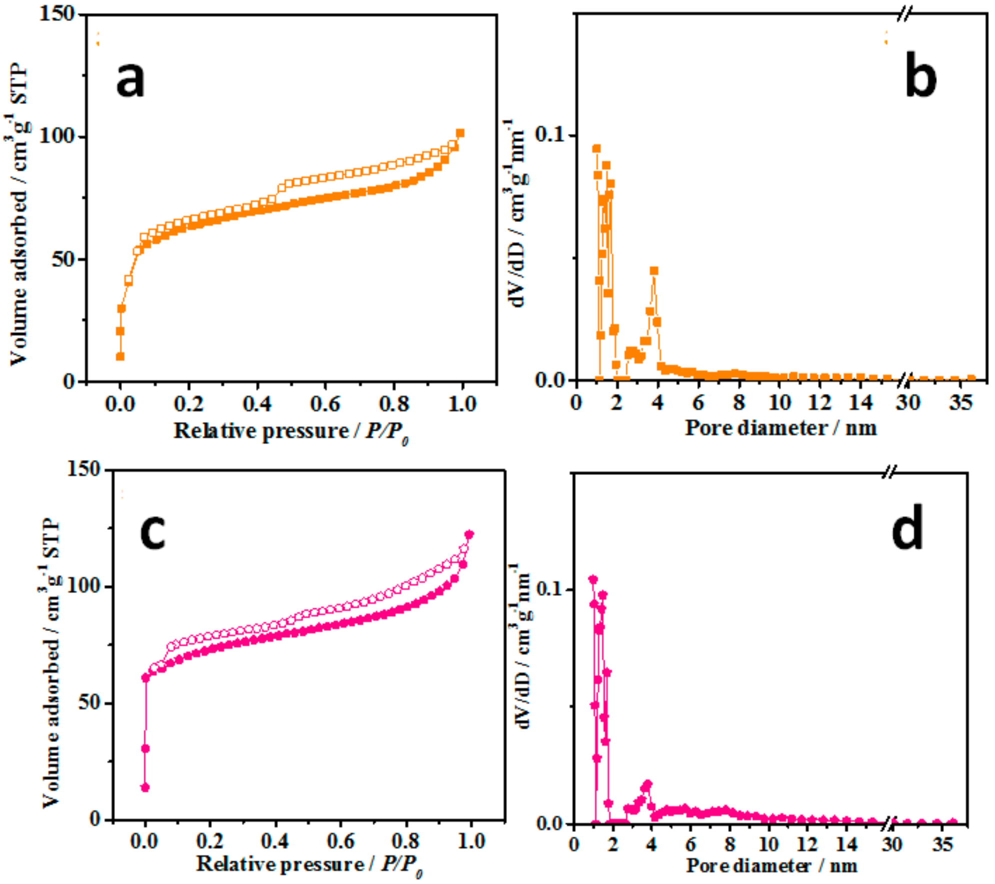

Figure S6. N_2 isotherms and pore size distributions of carbon materials of carbon materials obtained at (a,b) 300 °C and (c,d) 500 °C respectively.

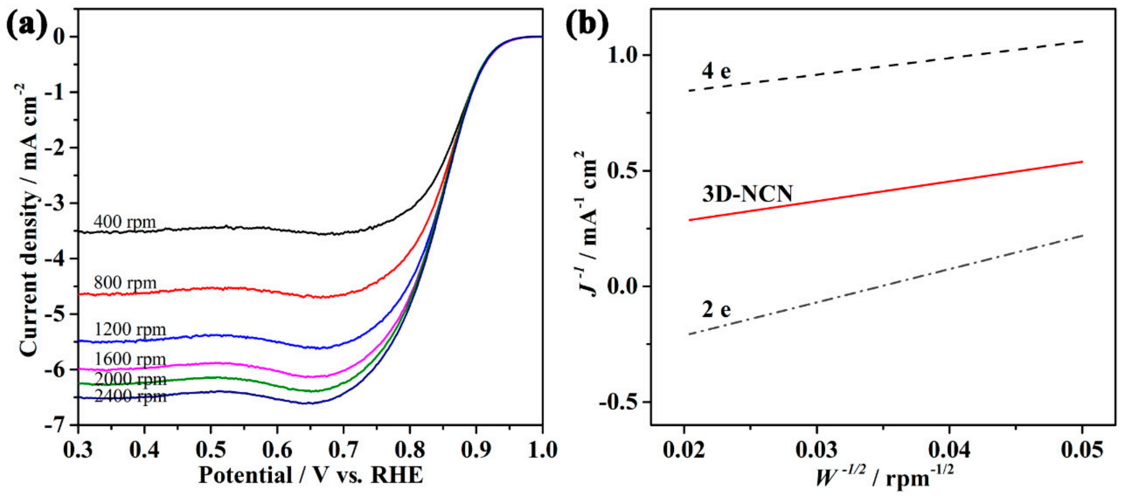

Figure S7. (a) Polarization curves of 3D–NCN in oxygen-saturated 0.1 M KOH solution at various rotation rate, scan rate is 5 mV s^{-1}. (b) Koutecky-Levich plots for 3D–NCN compared with ideal 2-electron and 4-electron processes at 0.462 V in 0.1 M KOH.
